# Improvement of a Real Gas-Sensor for the Origin of Methane Selectivity Degradation by µ-XAFS Investigation

**DOI:** 10.1007/s40820-015-0035-7

**Published:** 2015-02-17

**Authors:** Takahiro Wada, Naoyoshi Murata, Takuya Suzuki, Hiromitsu Uehara, Hiroaki Nitani, Yasuhiro Niwa, Motohiro Uo, Kiyotaka Asakura

**Affiliations:** 1 Graduate School of Medical and Dental Sciences, Tokyo Medical and Dental University, Tokyo, 113-8549 Japan; 2 Corporate R&D Headquarters, Fuji Electric Co., Ltd., Tokyo, 191-8502 Japan; 3 Catalysis Research Center, Hokkaido University, Sapporo, 001-0021 Japan; 4 Photon Factory, Institute of Materials Structure Science, KEK, Tsukuba, 305-0801 Japan

**Keywords:** Micro gas sensor, Micro-XAFS, Pd/Al_2_O_3_, Deactivation

## Abstract

We have directly investigated the chemical state of the Pd species in a real μ-gas sensor device by examining the μ-fluorescence X-ray absorption fine structure. The μ-gas sensor device was heavily damaged by a heating process in which the temperature was ill-controlled, resulting in decrease of methane selectivity. We found that the PdO in the fresh μ-gas sensor was reduced to Pd metal particles as the methane selectivity decreased. Based on the investigation results, we modified the device structure so as to heat up homogeneously. The lifetime of the sensor was then successfully increased by more than 5 years.

## Introduction

Natural gas is now used for household energy resources because of its ease of use and environmental friendliness. The development of safety measures, such as a gas leakage sensor is in strong demand. SnO_2_ is a kind of sensor materials, its electric conductivity changes with the gas composition at high temperature [1]. However, the requirement of the AC power supply hinders the daily use of the SnO_2_ gas sensor in the house because of the installation problem and the bad appearance.

**Fig. 1:**
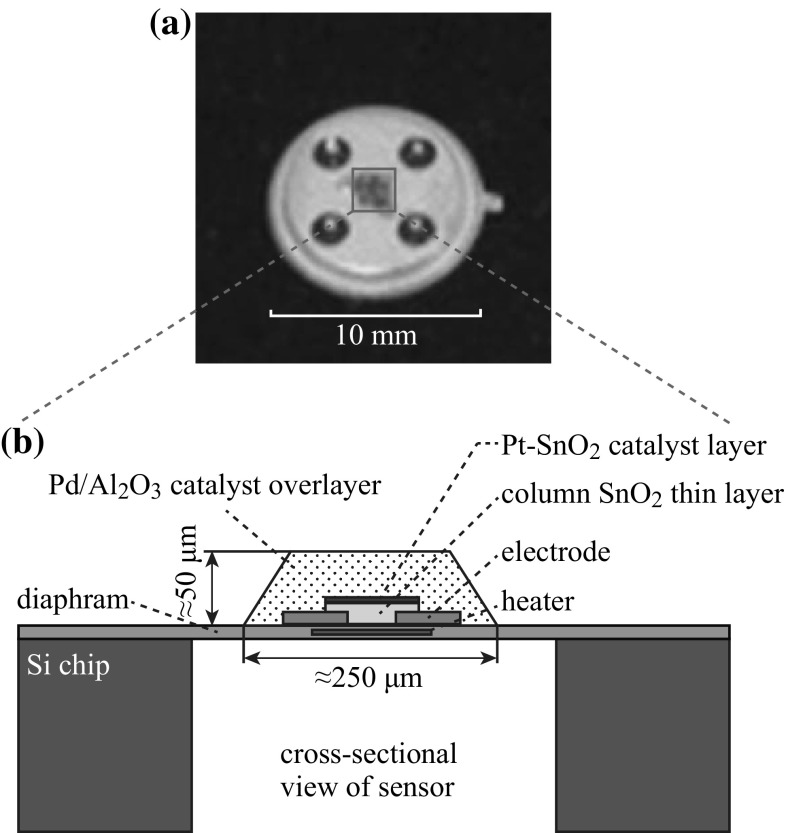
**a** Photograph of μ-gas sensor. **b** Diagram of μ-gas sensor chip cross section

Recently, Suzuki et al. have successfully developed a new SnO_2_ battery-driven sensor by a micro-electro-mechanical system (MEMS) method to achieve low power consumption [2, 3]. The key factors are the promoting catalysts and pulse-heating system. Figure 1 shows a schematic drawing of the μ-gas sensor chip involved. It is composed of a Pd/Al_2_O_3_ catalyst overlayer, Pt-SnO_2_ catalyst layer, SnO_2_ thin layer, electrode, and heater, which are sequentially deposited on a Si chip, giving the sensor high sensitivity and selectivity for methane. The SnO_2_ layer at the bottom was the heart of the sensor where the conductivity changes according to the concentration of the reductant gases. It was empowered by Pt/SnO_2_ layer with its sensitivity. Pd/Al_2_O_3_ increased the methane selectivity probably due to its high combustion activity of other reductant gases than methane [4]. A model sensor of a few square centimeter in area has been developed on a large Si substrate where the SnO_2_ thin layer, Pt-SnO_2_, and Pd/Al_2_O_3_ layers were deposited. Murata et al. characterized the structure of this model system using X-ray absorption fine structure (XAFS) and X-ray photoelectron spectroscopy (XPS). They revealed that the high sensitivity was due to the Pt^4+^ located in the SnO_2_ lattice [5]. However, in the real sensor device, the lifetime was not stable enough, especially in the selectivity for methane, although the sensitivity of the SnO_2_ thin layer was maintained. The selectivity instability was found to be related to the ill-controlled heating of the μ-gas sensor system. Since Pd/Al_2_O_3_ is responsible for the selectivity and the damaging process can hardly be reproduced exactly in a model large sensor system, we directly investigated the structure of the Pd/Al_2_O_3_ layer in a real, small μ-gas sensor, the diameter of which was about 250 μm, before and after the damaging process. Therefore, the measurement of this sample requires μm order spatial-resolved XAFS, so called μ-XAFS. We used a μ-XAFS method to investigate the origin of the decrease in selectivity. We used a polycapillary lens to focus the X-ray into a μ-beam [6–9]. The μ-X-ray size was smaller than the sensor’s Pd/Al_2_O_3_ area. We could monitor the structure change of the μ-gas sensor. In this paper, we discuss the possible origin for the decreases in the methane selectivity of the μ-gas sensor together with the role of the Pd/Al_2_O_3_ layer and the importance of homogeneous heating in the gas sensor.

## Experimental

### μ-XAFS

Pd K-edge μ-XAFS experiments were performed at the NW-10A beamline at the Photon Factory (Institute for Materials Structure Science, High Energy Accelerator Research Organization; KEK-IMSS-PF) using a Si (311) double crystal monochromator in a fluorescence mode [10]. The original beam size of this beamline was 1 × 1 mm^2^.

**Fig. 2:**
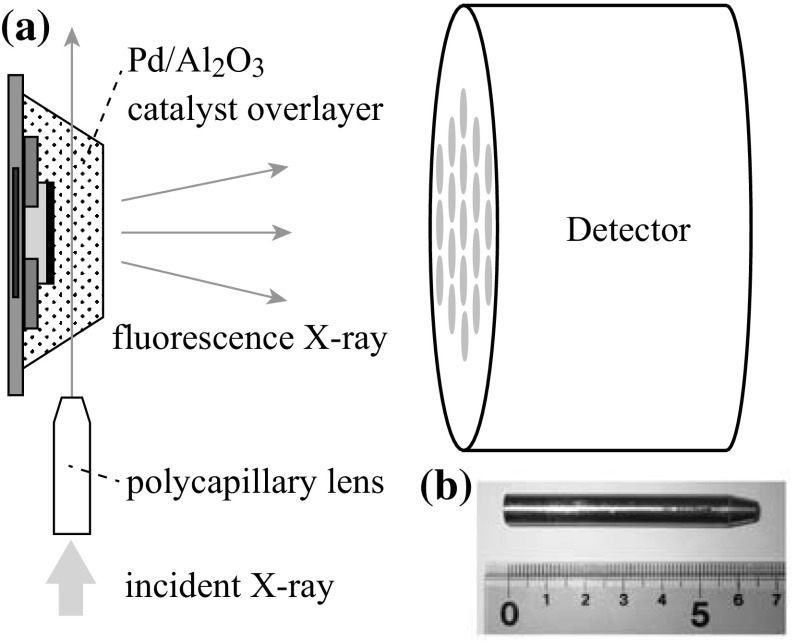
**a** Alignments of μ-beam and μ-gas sensor. The sample surface was mounted roughly parallel to the X-ray incident direction and the detector surface. The cross section area of the X-ray is 25 μm in FWHM diameter as measured by a knife-edge scan. **b** Photograph of polycapillary lens

A polycapillary lens is used in such microanalysis techniques as X-ray fluorescence analysis (XRF) [11–14], XAFS [15–19], and photoemission electron microscopy (PEEM) [20]. Some pioneers reported confocal measurement will give the 3D resolved point analysis using two capillaries or a combination of capillary and other x-ray optics [21–26]. The X-ray beam was focused by a polycapillary lens having a focal distance of 9.5 mm and transmission efficiency of about 8 % at 25 keV (XOS Inc., USA). The experimental setup is given in Fig. 2. The incident X-ray beam intensities were monitored by a 170 mm-long ionization chamber. The X-ray fluorescence was detected by a 19-element Ge-SSD (Ortec, USA). The focal spot size was about 25 μm in diameter (FWHM) as measured by knife-edge scans. XAFS spectra were analyzed using REX2000 software (Ver. 2.5, Rigaku, Japan) [27].

### μ Gas Sensor

**Fig. 3:**
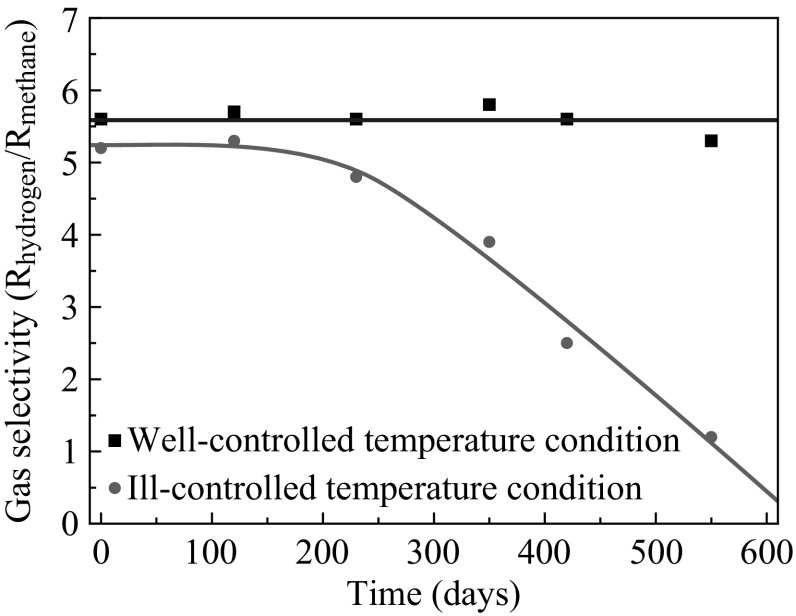
Test results for accelerated aging of the samples subjected to well-controlled (*square*) and the ill-controlled (*circle*) temperature conditions

The μ-gas sensors were prepared as described elsewhere [2, 3, 5, 28, 29]. Figure 1 shows the structure of the μ-gas sensor. The Pd/Al_2_O_3_ thickness was about 50 μm. We set the X-rays parallel to the sensor base, as shown in Fig. 2, where the maximum fluorescence X-ray intensity was obtained. The methane selectivity of the sensor was decreased when the sensor underwent ill-controlled temperature treatment where the heating temperature varied as 653 ± 50 K. When the temperature was well-controlled at 703 K, the decrease in the selectivity was not observed, as shown in Fig. 3. The sample was treated to obtain a damaged sample for the acceleration aging test, where the μ-gas sensors were driven in a flow of 500 ppm H_2_ (a model reductant gas), and 99 % relative humidity (RH). Selectivity (*Sel*) was defined as follows:

$$ Sel = R_{{_{hydrogen} }} /R_{{_{methane} }} $$

where *R*_*hydrogen*_ and *R*_*methane*_ are the resistance in flow of 1000 ppm H_2_ and 1000 ppm CH_4_ at 20 °C and 65 % RH. Four typical examples *Sel* = 5.2 (Fresh), *Sel* = 3.7 (*Sel*3.7), *Sel* = 1.8 (*Sel*1.8), and *Sel* = 0.9 (*Sel*0.9) were measured.

## Results and Discussion

**Fig. 4:**
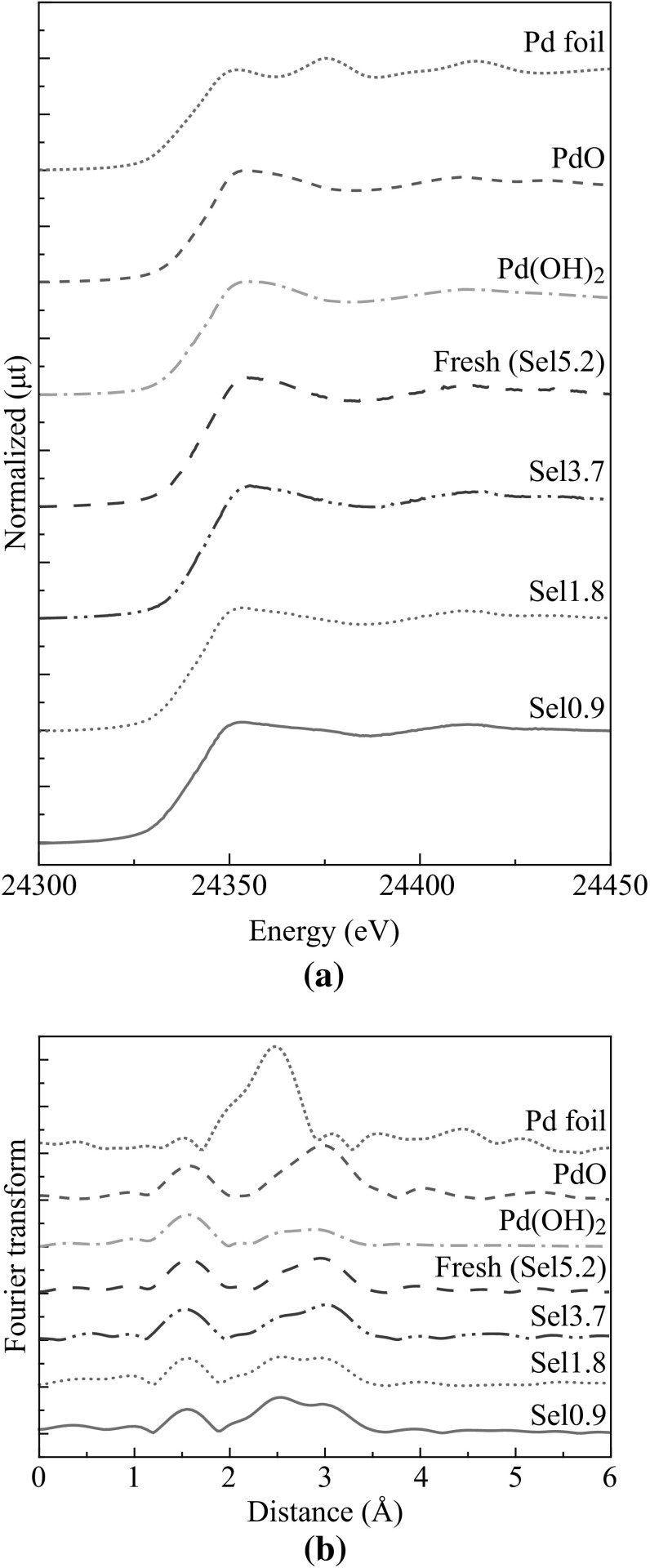
**a** Pd K-edge XANES spectrum. **b** Pd K-edge FT-EXAFS

**Fig. 5:**
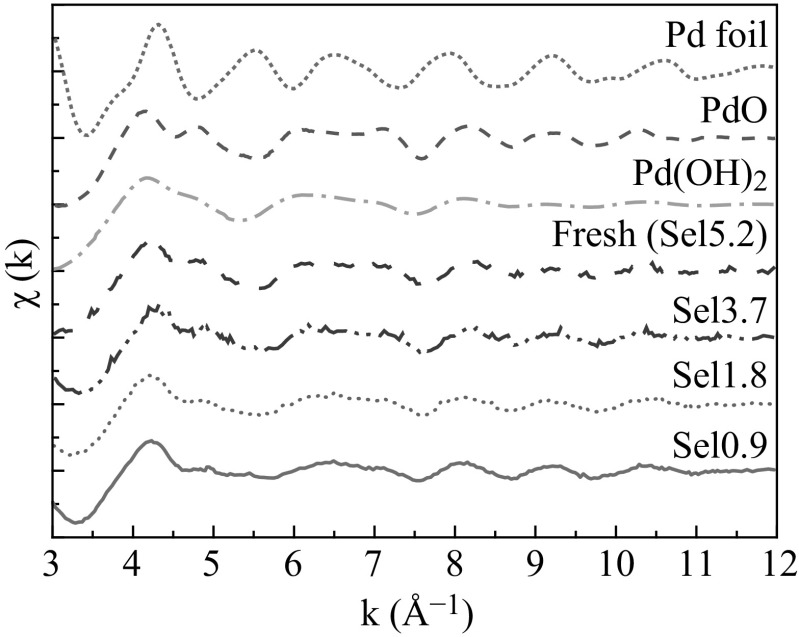
Pd K-edge EXAFS spectrum of real sensor head measured by polycapillary

Figure 4 shows the Pd K-edge XANES (Fig. 4a) and EXAFS (Fig. 4b) for fresh and treated samples. We could obtain enough data for analysis as shown in Fig. 5, even if the sample thickness was only 50 μm using a polycapillary tube.

The XANES spectrum in the fresh sample was much more similar to those of PdO and Pd(OH)_2_ rather than Pd foil. When the methane selectivity was decreased, the XANES changed a little. The first edge peak (24,350 eV) decreased with the increase in the higher energy side (24,360 eV) as the methane selectivity decreased. EXAFS Fourier transforms provided more definite structural information. In the Fourier transform of the fresh sample, Pd–O was found at 0.16 nm together with Pd–Pd at about 0.3 nm, indicating the formation of PdO, not Pd(OH)_2_. As the methane selectivity decreased, we found a new, emerging peak at 0.25 nm in the Fourier transform. This new peak could be assigned to the Pd–Pd bond in the Pd metal. The height of this peak increased with the decrease in methane selectivity. Curve fitting results indicated the presence of Pd at 0.273 ± 0.004 nm with a coordination number of 2.8 ± 0.6 in *Sel*0.9. In *Sel*3.7 and *Sel*1.8, we found the coordination number of the Pd–Pd bond in Pd metal to be 0.9 ± 0.4 and 2.4 ± 1.0, respectively.

**Fig. 6:**
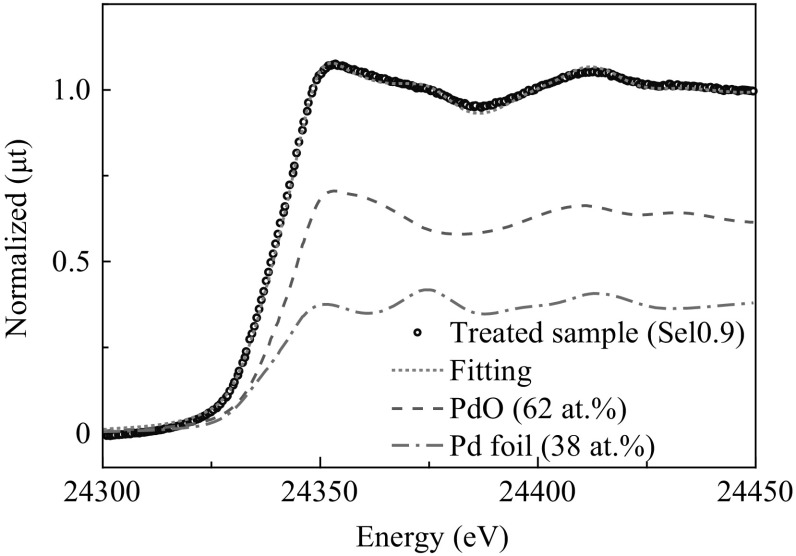
Linear combination fitting results using XANES spectra of Pd foil and PdO. The XANES spectra were of a treated sample (*Sel*0.9) (*open circle*). **a** Fitting results combined **b** PdO (62 at.%) and **c** Pd foil (38 at.%)

The decrease in the methane selectivity seemed to be strongly correlated with the formation of Pd metal particles. We assumed that the change of XANES was due to the increase in Pd metal concentration in the composition. We carried out a linear combination analysis using XANES spectra of Pd foil and PdO. Figure 6 shows the fitting results for the *Sel*0.9 sample. The data were well reproduced using spectra of metallic Pd (38 at.%) and PdO (62 at.%).

**Fig. 7:**
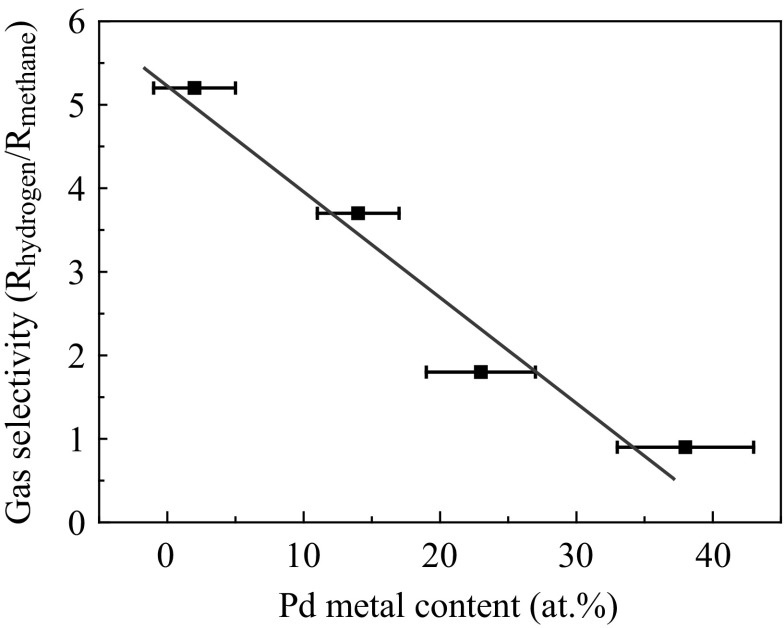
Relation between the methane selectivity and PdO content

The decrease in the selectivity was negatively proportional to the Pd metal content, as shown in Fig. 7. Errors of Pd metal content were estimated using the Hamilton ratio method [30] at a confidence level of 95 %. Therefore, the selectivity decrease occurred due to the formation of Pd particles.

Previous research has shown that the function of the Pd/Al_2_O_3_ catalyst is to improve the selectivity for methane [4]. Methane is inert compared to the other gases that are present. The PdO in the Pd/Al_2_O_3_ layer can burn up the other gases (hydrogen, alcohol, carbon monoxide, etc.) completely, but not the methane that reaches the sensor part (SnO_2_ thin layer). The formation of Pd changes the activity and selectivity of the Pd/Al_2_O_3_ overlayer. Consequently, the selectivity decreases. Pd and PdO have different activation behaviors for hydrogen and other gases [31]. The PdO is necessary for the selective combustion and increases the selectivity.

Under ill-controlled temperature conditions, the Pd was not always heated up to 703 K. In the model system, we found the PdO was reduced at intermediate temperatures (500–600 K) in the presence of H_2_ and reoxidation of Pd metal occurred at higher temperatures [31]. Therefore, insufficient heating may sometimes create Pd particles, which may aggregate. Once the Pd is aggregated into large metal particles, high-temperature heat treatment cannot redisperse the Pd particles to the PdO again, even at 703 K. In the sample that was subjected to ill-controlled temperature conditions, the aggregation to form large Pd particles might occur slowly but steadily, causing the sensor to gradually lose its selectivity. Moisture also accelerates the aggregation process [4]. Under the influence of moisture, the surface of PdO might consist of Pd(OH)_2_, which should show high mobility and thus accelerate the aggregation of large Pd particles [32, 33]. In the dry gas, the acceleration rate of the decreasing selectivity was low. The heating treatment at 703 K also helps the removal of surface Pd(OH)_2_ species. We conclude the PdO is the key factor in keeping the high selectivity for methane. The Pd/Al_2_O_3_ layer of micro gas sensors structure was modified to increase the heat retention capability and the contact area to the heater and to decrease the thermal capacity. As results, it allowed that the temperature was kept around 703 K homogeneously without increasing power consumption and PdO structure can be maintained. Based on the knowledge obtained here, the battery-driven μ-sensor has been realized to attain the enough lifetime more than 5 years with the sensor structure to heat the sensor homogeneously.

## Conclusion

In this work, an X-ray μ-beam made by a polycapillary was used to measure the μ-XAFS of a model μ-gas sensor. These results suggest that the ill-controlled heating of the μ-gas sensor system caused the reduction of PdO to Pd metal particles at medium temperature. Since the Pd nanoparticle was active for the oxidation reaction of methane, the methane selectivity decreased. However, at a higher temperature than 703 K, the Pd was kept in PdO structure even in the presence of reductant gas (H_2_), indicating that the homogeneous heating of the sensor is essential to keep the PdO structure. Based on this result, the μ-gas sensor structure has been modified to keep the sensor device at the high temperature homogeneously, and the sensor lifetime has successfully been increased by more than 5 years. The μ-XAFS is a powerful analytical tool that gives important information in understanding the mechanism of real devices at the atomic level.
